# Quantitative electroencephalogram study in patients with schizophrenia : a literature review

**DOI:** 10.1192/j.eurpsy.2023.592

**Published:** 2023-07-19

**Authors:** D. Jardak, N. Smaoui, L. Triki, M. Mnif, I. Gassara, R. Feki, M. Maalej, N. Charfi, J. Ben thabet, L. Zouari, S. Omri, M. Maalej

**Affiliations:** psychiatry, Hedi Chaker Sfax Hospital, sfax, Tunisia

## Abstract

**Introduction:**

There has been a continuous effort to discover and specify the neural correlates of schizophrenia (SCZ) based on spontaneous electroencephalogram (EEG) records. Besides contributing to a more effective diagnosis, biomarkers can be crucial to SCZ to hope for therapeutic progress.

**Objectives:**

a literature review was conducted to ascertain whether or not quantitative EEG spectral abnormalities are consistent enough to warrant additional effort towards developing them into a clinical diagnostic test for schizophrenia.

**Methods:**

A systematic search of the databases , ScienceDirect, and PubMed was conducted using the following words : schizophrenia, electroencephalography, neurobiology. The Preferred Items Reporting for Systematic Reviews and Meta-Analyses (PRISMA) guidelines were followed in the construction of this literature review. Primary research articles that reported descriptive EEG results, included comparisons of subjects with and without antipsychotic therapy, and excluded patients with epilepsy were included in the analysis. We analyzed pooled data, where possible, from studies with a similar intervention and methodology.

**Results:**

Our study included 11 articles on quantitative EEG changes in schizophrenic patients divided as follows: 2 articles on the genetics of SCZ and EEG data, 3 articles on the psychopathology of SCZ and EEG data, 2 articles on hemispheric coherence, and finally 4 articles on the effect of treatment on EEG.Increased beta activity can be considered as an inherited feature of SCZ. Elevated delta/theta and gamma activity may serve as a specific biomarker for this condition. The delta wave may be a neurophysiological tool to differentiate between negative and positive forms of SCZ.EEG tracings of schizophrenic patients showed increased intra- and inter-hemispheric coherence compared to healthy subjects. Treatment with an antipsychotic drug was associated with a more marked increase in frequency bands in patients receiving an atypical antipsychotic drug.

**Conclusions:**

It is important to study the electroencephalographic changes not only to better understand the etiopathogenesis of SCZ, but also to search for specific physiological biomarkers.

**Disclosure of Interest:**

None Declared

